# Multiplex Biomarker Screening Assay for Urinary Extracellular Vesicles Study: A Targeted Label-Free Proteomic Approach

**DOI:** 10.1038/s41598-018-33280-7

**Published:** 2018-10-09

**Authors:** Somchai Chutipongtanate, Kenneth D. Greis

**Affiliations:** 1 UC Proteomics Laboratory, Department of Cancer Biology, University of Cincinnati College of Medicine, Cincinnati, Ohio, United States; 2 Pediatric Translational Research Unit, Department of Pediatrics, Faculty of Medicine Ramathibodi Hospital, Mahidol University, Bangkok, Thailand

## Abstract

The recent advance in targeted label-free proteomics, SWATH-MS, can provide consistent protein detection and reproducible protein quantitation, which is a considerable advantage for biomarker study of urinary extracellular vesicles. We developed a SWATH-MS workflow with a curated spectral library of 1,145 targets. Application of the workflow across nine replicates of three sample types (exosome-like vesicles (ELVs), microvesicles (MVs) and urine proteins (UPs)) resulted in the quantitation of 888 proteins at FDR <1%. The median-coefficient of variation of the 888 proteins in the ELV sample was 7.7%, indicating excellent reproducibility. Data analysis showed common exosome markers, (i.e. CD9, CD63, ALIX, TSG101 and HSP70) were enriched in urinary ELVs as compared to MVs and UPs. The use of a multiplex biomarker screening assay focused on ELVs was investigated, and perspectives in future applications are discussed.

## Introduction

Since their discovery by Pisitkun T, *et al*.^1^, urinary extracellular vesicles have become one of the most promising reservoirs of biomarkers in kidney diseases. Urinary extracellular vesicles contain exosomes (or exosome-like vesicles (ELVs)), microvesicles (MVs) and apoptotic bodies, all of which are distinguishable by their physical and biochemical properties^1–4^. Proteomics could play a remarkable role in urinary ELV study; however, consistency and reproducibility of data obtained from label-free data-dependent acquisition (DDA) have remained a challenge. Classical targeted proteomics (i.e., multiple reaction monitoring, MRM) has limited multiplexing capability (10s-100s) and requires costly isotope standards.

SWATH-MS (*s*equential *w*indow *a*cquisition of all *th*eoretical fragment ion-*m*ass *s*pectrometry)^5^ improves consistency and reproducibility of protein detection and achieve highly multiplexing, MRM-like, label-free protein quantitation (up to 100s-1000s) by data-independent acquisition (DIA) with spectral library-based data extraction^6,7^. This approach also opens a possibility to update and customize the spectral library for long-term use in particular projects.

This article communicates a development of SWATH-MS that is optimized for urinary ELV analysis, an ELV-SWATH-MS workflow. Performance data and an example of analyses are shown, while a perspective on potential applications is provided.

## Results and Discussion

**Figure 1:**
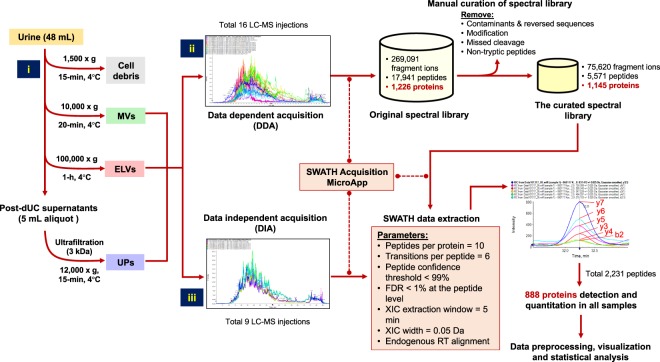
The schematic diagram represents the entire process of ELV-SWATH-MS workflow. Three main steps included; (i) the isolation of urinary extracellular vesicles including exosome-like vesicles (ELVs) and microvesicles (MVs) by differential ultracentrifugation and subsequently concentrating urine proteins (UPs) by ultrafiltration (3-kDa cutoff); (ii) the spectral library generation based on all identified fragment ions, peptides and proteins from data-dependent acquisition (DDA) of 16 protein fractionations of ELVs, MVs and UPs and a manual curation to remove the sources of variability in targeted proteomic analysis; (iii) SWATH analysis using predefined targets in the curated spectral library for targeted data extraction and peptide quantitation from the digital protein records of ELVs, MVs and UPs generated by data-independent acquisition (DIA).

**Figure 2:**
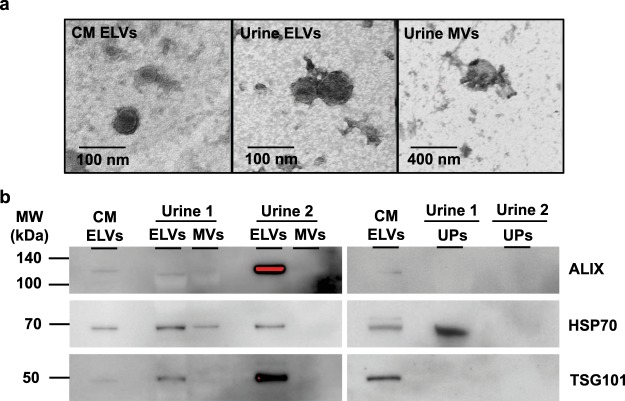
Validation of ELVs presence by particle and protein analyses. (**a**) Transmission electron microscopy (TEM) with the negative staining showed cup-shaped vesicles with a diameter less than 100 nm resembling exosomes presented in the ELV sample, whereas the vesicles larger than 100 nm were observed in the MV sample. Magnification power of 100,000x and 400,000x were applied for the MV and ELV samples, which corresponding to scale bars of 400 nm and 100 nm, respectively. (**b**) Western blot analysis showed enrichment of three exosome markers (i.e., ALIX, TSG101 and HSP70) in urinary ELVs as compared to urinary MVs and UPs isolated from two independent experiments. ELVs separated from culture media (CM) of MOLM13 cells served as the positive control in validation experiments. The red color in the immunoreactive band indicated the saturated signal intensity. The full-length blots of three exosome markers were provided in Supplementary Figure S1.

The entire process of the ELV-SWATH-MS workflow is shown in Fig. 1. The enrichment of ELVs was accomplished through differential ultracentrifugation (dUC), and validation for the presence of ELVs by transmission electron microscopy (TEM) to confirm the vesicular diameter in the range of 40–100 nm^1–4^ and by Western blotting for three exosome markers^2^ including programmed cell death-6 interacting protein (PDCD6IP; commonly known as ALIX), tumor susceptibility gene 101 protein (TSG101) and heat shock protein 70 (HSP70) (Fig. 2a and b). Additional characterization using a nanoparticle tracking analysis also supported the presence of ELVs in the isolates (Supplementary Figure S2). These criteria were consistent with the reported standard for enriched ELVs^8,9^ and thus supported the use of these ELVs for further evaluation in the ELV-SWATH-MS workflow.

### Generation of a curated spectral library of 1,145 proteins

To build the spectral library, 16 fractions consisting of urine proteins (UPs), ELVs and MVs were separated and identified by nanoLC-MS/MS followed by a database search using standard shotgun proteomics (or DDA methods)^5–7^. An original spectral library was built upon all fragment ions, peptides and proteins identified from 16 fractions (Fig. 1). After manual curation to remove sources of variability (i.e. modification, missed cleavage, or non-tryptic peptides), the final curated spectral library contained 1,145 proteins, covering 93% of proteins in the original library (Fig. 1). Functional annotation revealed 947 out of 1,145 proteins associated with extracellular exosomes (adjusted *p*-value < 1.00E-99) (Supplementary Table S1). The curated spectral library was used for further SWATH data extraction.

### Performance of the ELV-SWATH-MS workflow

**Figure 3:**
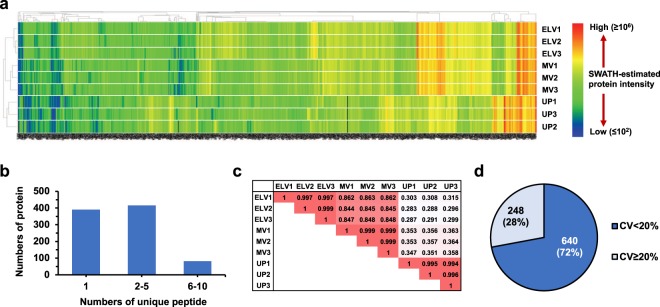
Performance of the ELV-SWATH-MS workflow. (**a**) Quantitative data of 888 proteins in ELVs, MVs and UP samples (3 replicates for each sample) were demonstrated in a heat map manner. The scale of SWATH-estimated protein intensity showed the relative amount of original data after Total Area Sum (TAS) normalization. The missing values (6 out of 7,992 data across all replicates) were labeled in black color. (**b**) Numbers of unique peptide per protein. (**c**) Pearson correlation of nine sample replicates based on 888 expression data. Each number in the sample matrix was the correlation coefficient (*r*), in which *r* = 1 was a perfect positive relationship, and *r* = 0 showed no association between a sample pair. (**d**) The coefficient of variation (CV) of 888 protein quantitation in urinary ELVs, in which the median-CV was 7.7%.

SWATH-MS analysis provided excellent expression data with a great depth of proteome coverage as demonstrated by 888 proteins detected and quantified at FDR <1% across all samples (ELV, MV and UP samples; 3 replicates each), in which only 6 out of 7,992 data points were missing (Fig. 3a and Supplementary Table S2). It should be noted that not all proteins surviving the threshold of FDR <1% were confidently identified, and at this threshold up to 9 out of 888 proteins were possibly false positive results. The majority of proteins (497 out of 888) were measured based on >2 unique peptides per protein (Fig. 3b**)**. Pearson analysis showed excellent correlation among 3 ELV replicates (average *r* = 0.998), high between ELV-MV replicates (average *r* = 0.851), but poor between ELV-UP replicates (average *r* = 0.296) (Fig. 3c). Moreover, the median-coefficient of variation (CV) of all protein quantitation in the ELV sample was 7.7%, and 640 out of 888 assays had the CV <20% (Fig. 3d) which was acceptable for the highly multiplex proteomic assay^7,10^. This performance data supported further applications of the ELV-SWATH-MS workflow for urinary ELVs study.

**Figure 4:**
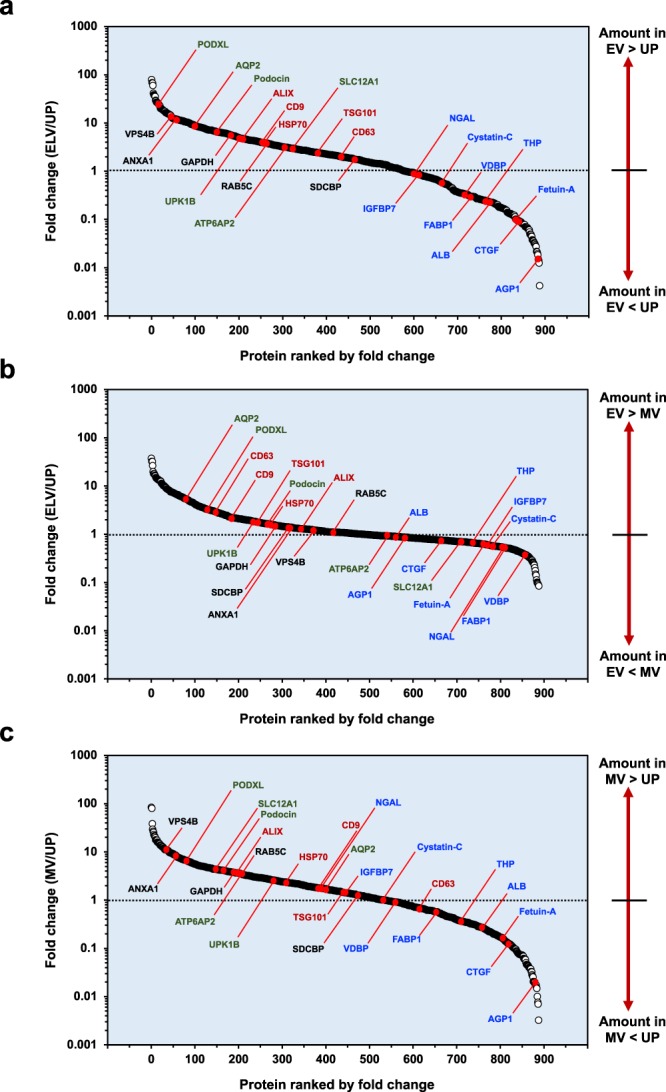
Protein enrichment analysis. Mean values of protein expression was used to calculate fold change (FC) of particular proteins between ELV-UP (**a**), ELV-MV (**b**) and MV-UP (**c**), in which FC >1, <1, and = 1 indicated enrichment, depletion and no change, respectively. Selected biomarkers were labeled to facilitate visual interpretation. Common exosome markers were marked in red color. Tissue-localized markers were labeled in green color. Exosome-associated proteins were labeled in black color. Selected urine biomarkers were labeled in blue color.

Next, we evaluated the dynamic ranges of fold-changes between ELV-UP, ELV-MV and MV-UP pairs for the 888 proteins (Fig. 4). Selected biomarkers^2–4,11^ were labeled to facilitate visual interpretation, including common exosome markers (i.e., CD9, CD63, ALIX, TSG101, HSP70), tissue-localized markers (i.e., podocin and podocalyxin (PODXL) from glomerular podocytes, (pro)renin receptor (ATP6AP2) from glomerular mesangium, sodium-potassium-chloride cotransporter (SLC12A1) from thick-ascending limb of Henle’s loop, aquaporin 2 (AQP2) from collecting duct, and uroplakin-1B (UPK1B) from bladder urothelium), exosome-associated proteins (e.g., annexin A1 (ANXA1), vacuolar protein sorting-associated protein 4B (VPS4B), syntenin-1 (SDCBP), Ras-related protein Rab-5C (RAB5C), glyceraldehyde-3-phosphate dehydrogenase (GAPDH), and selected urine biomarkers (i.e., albumin (ALB), Tamm-Horsfall protein (THP; also known as uromodulin), α1-acid-glycoprotein 1 (AGP1), fetuin-A, insulin-like growth factor-binding protein 7 (IGFBP7), neutrophil gelatinase-associated lipocalin (NGAL), cystatin-C, vitamin-D binding protein (VDBP), fatty acid binding protein, liver type (FABP1) and connective tissue growth factor (CTGF)). As expected, common exosome markers were enriched in the ELVs as compared to the UP and MV samples, while the opposite was true, for urine biomarkers (Fig. 4a and b). Common exosome markers were presented in MVs, but a much lower levels than those of ELVs (Fig. 4c). This result was consistent with the correlation data (Fig. 3c), suggesting that both vesicle samples shared some proteins and characteristics^4,12^, but this could also be due to entrapment of ELVs in the low-speed MV pellet^13^.

Note that ALIX and TSG101 in the MV and UP samples were not identified by Western blotting (Fig. 2b), but were by SWATH (Fig. 4). It is not uncommon that low abundance proteins that are measurable by mass spectrometry-based methods to not be detected by Western blot. For a given targeted protein, it is known that a linear dynamic range and a limit of detection of Western blot analysis are variable depending on several factors including the performance of antibodies, signal enhancing systems, and exposure-time durations. While mass spectrometry-based targeted proteomics, including SWATH, measure absolute amounts of peptide masses corresponding to a targeted protein and thus can demonstrate a lower limit of detection (fmol-to-amol levels) and the wider linear dynamic range (up to six orders of magnitude)^7^. In contrast, a very high affinity antibody with minimal cross-reactive coupled with the signal amplification using luminescent reagents can sometime provide better lower limits of detection than mass spectrometry. In the end, the detection limits for a given protein are protein dependent based on the antibody available and the peptides that can be generated for mass spectrometry. Overall however, the performance characteristics of modern mass spectrometry-based methods often outperform Western blotting.

### Application potential

**Figure 5:**
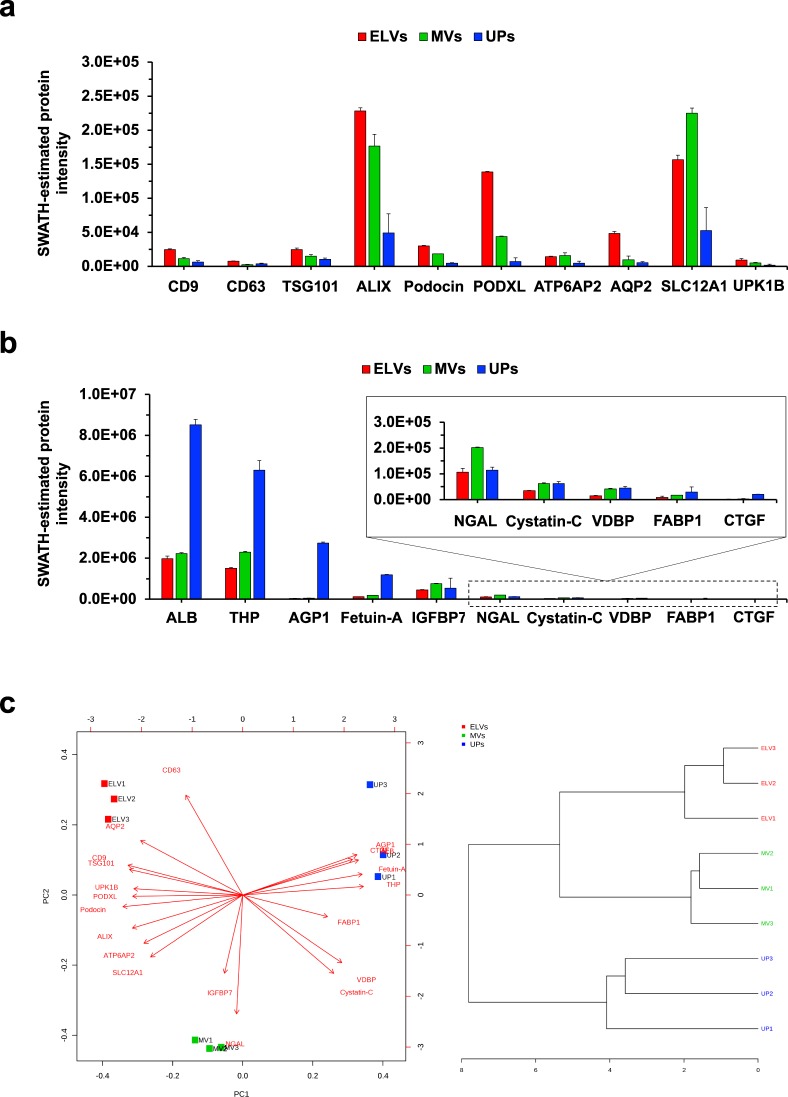
A potential application of the ELV-SWATH-MS workflow as a multiplex biomarker screening assay. A panel of 20 biomarkers consisting of proteins related to urinary extracellular vesicles (**a**) and candidate urine biomarkers (**b**) was applied for targeted data extraction of ELVs, MVs and UP samples. Based on expression data in this panel, three ELV replicates were classified out of the MV and UP samples by unsupervised principal component analysis (left panel) and hierarchical clustering (right panel) (**c**). This result supported future development of multiplex biomarker screening assay as a part of quality control in urinary ELV isolation.

To highlight the potential applicability of the ELV-SWATH-MS workflow in a hypothesis-driven manner, we asked whether a panel of selected biomarkers (as presented in Fig. 4) in conjunction with machine learning algorithms could distinguish ELVs from MVs and UPs. Figure 5a and b demonstrated expression data of 20 biomarkers in the panels, in which CD9, CD63, TSG101, ALIX and most tissue-localized proteins were higher and urine biomarkers were lower in ELVs as compared to MVs and UP samples. Based on this panel, unsupervised principal component analysis and hierarchical clustering (Fig. 5c) could distinguish three ELV replicates apart from the MV and UP samples as expected. This data was an example of analyses using a small sample size. Nonetheless, it provided a vivid image of how to apply the ELV-SWATH-MS workflow in hypothesis-driven projects. Future development of multiplex biomarker panels based on the ELV-SWATH-MS workflow coupled with machine learning algorithms may be implemented as a quality control of urinary ELV isolation.

Going forward, the ELV-SWATH-MS workflow should offer the flexibility to address different problems related to urinary ELVs in both discovery and targeted approaches. The spectral library can be updated for new targets from local experiments, and possibly, retrieved from public databases, i.e., SRMAtlas (www.srmatlas.org)^14^ and SWATHAtlas (www.swathatlas.org)^15^, and then customized to meet specific needs of particular projects. Recently, Kulkarni S and colleagues^16^ reported the first SWATH application to discover 23 candidate biomarkers of radiation exposure-induced injury in mouse urinary exosomes. To evaluate this potential finding in a clinical setting, the spectral library can be customized for a robust and cost-effective screening of radiation injury signatures in patient-derived urinary exosomes using the ELV-SWATH-MS workflow. By affinity-based methods^2,17^, ELVs originating from particular nephron segments may be isolated explicitly for SWATH-MS analysis, and thus may expand a working definition of liquid biopsy. A significant question in urinary ELV study is what the most suitable normalization method. The ELV-SWATH-MS workflow may be applied to search for “a time-constant marker” in lieu of time normalization based on the gold standard 24-h urine (Pisitkun T and Mischak H; *Personal Communication*). These ideations should be explored for future feasibility.

In conclusion, the ELV-SWATH-MS workflow has been demonstrated to provide significant depth of proteome coverage with a high level of quantitative reproducibility. Further optimization and application to a variety of clinical maladies for kidney diseases may allow for new and robust avenues for biomarker identification and validation for clinical use.

## Materials and Methods

### Urine sample

All methods were carried out in accordance with relevant guidelines and regulations. Urine samples (2 healthy subjects, male gender, age 59 and 61 years) were purchased from the University of Cincinnati (UC) Biorepository as de-identified, banked specimens and thus did not require human subject IRB approval. Each 50-mL urine sample was added 1 tablet of protease inhibitor cocktail (Roche Diagnostics GmbH, Mannheim, Germany) and kept at −80 °C until used.

### Sample preparation

MVs and ELVs were consecutively isolated from urine (48-mL) by dUC at 10,000 × g, 4 °C, 20-min and at 100,000 × g, 4 °C, 1-h (OptimaXE-100, Beckman Coulter, IN; SW60Ti rotor; k-factor 107.36). UPs were concentrated by 3-kDa ultrafiltration (Millipore Sigma, Burlington, MA). ELVs isolated from MOLM-13 culture media (CM; 100-mL) served as the positive control. Vesicle pellets were solubilized by RIPA buffer containing 60 mg/mL dithiothreitol with sonication (30% amplitude, 3-sec for five times) for protein analysis. Protein concentration was determined by Pierce660 assay (ThermoFisher, Florence, KY).

### Transmission electron microscopy (TEM)

TEM was used to verify the presence of extracellular vesicles, i.e, ELVs and MVs in the isolates. Five microliters of vesicle sample were applied to a formvar/carbon coated-copper 200 mesh grid (FCF200-CU; Electron Microscopy Sciences, Hatfield, PA) for 5 min at room temperature. The adsorbed vesicles were negatively stained with 2% uranyl acetate for 2 min and then dried at room temperature. The vesicle sample was examined by a JEM-1230 electron microscope (JEOL USA, Inc., Peabody, MA).

### Western blot analysis of three common exosome markers

Proteins (10 μg) were separated by SDS-PAGE and transferred to a polyvinylidene difluoride membrane. After blocking with 5% skim milk, immunodetection was performed by anti-ALIX (1:1000) (#2171 S; Cell Signaling Technology, Inc., Danvers, MA), anti-TSG101 (1:1000) (#sc-7964; Santa Cruz Biotechnology, Inc., Dallas, TX) and anti-HSP70 (1:1000) (#MA3-009; ThermoFisher) monoclonal antibodies at 4 °C overnight. The membrane was washed and then incubated with anti-mouse HRP-conjugated secondary antibody (1:5000) (#NA931; GE Healthcare, Pittsburgh, PA) at room temperature for 1 h. The membrane was incubated with enhanced chemiluminescence reagent (Immobilon; MilliporeSigma, Burlington, MA), followed by detection with ChemiDoc Touch Imaging system (BioRad Laboratories, Inc., Hercules, CA).

### Mass spectrometry

Nanoliquid chromatography coupled to electrospray tandem mass spectrometry (nanoLC-ESI-MS/MS) analysis was performed by a TripleTof 5600 + mass spectrometer (Sciex; Concord, Ontario, Canada) coupled with a nanoLC-ultra nanoflow system (Eksigent, Dublin, CA) in DDA or DIA modes^18^. After in-gel tryptic digestion, an amount of peptide sample corresponding to 2.5 μg of total protein was load via an Eksigent NanoLC-AS-2 autosampler onto a column trap (Eksigent Chrom XP C18-CL-3 µm 120 Å, 350 µm x 0.5 mm; Sciex, Toronto, Canada) at 2 µL/min in 0.1% formic acid for 15 min to desalt and concentrate the sample, which subsequently submitted into Acclaim PepMap100 C18 LC column (75 µm x 15 cm, C18 particle sizes of 3 µm, 120 Å) (Dionex; Thermo Fisher Scientific, Inc.) for chromatographic separation. The peptides were eluted at a flow rate of 300 nL/min using a variable mobile phase (MP) gradient from 95% phase A (0.1% formic acid) to 40% phase B (99.9% acetonitrile in 0.1% formic acid) for 70 minutes, from 40% phase B to 85% phase B for 5 minutes, and then keeping 85% phase B for 5 minutes. The nanoLC eluate was ionized and sprayed into the mass spectrometer using NANOSpray III Source (Sciex). Ion source gas 1 (GS1), ion source gas 2 (GS2) and curtain gas (CUR) were respectively kept at 13, 0 and 35 vendor specified arbitrary units. Interface heater temperature and ion spray voltage were kept at 150 °C and at 2.6 kV, respectively.

#### Data-dependent acquisition (DDA) mode

DDA method was operated in positive ion mode set to go through 1,929 cycles for 90 minutes, where each cycle performed 1 time of flight (TOF) mass spectrometry scan type (250 ms accumulation time, 350–1250 m/z window with a charge state of 2+ to 4+) followed by information dependent acquisition of the most 50 intense candidate ions. The minimum MS signal for triggering MS/MS scan was set to 150 counts. Each MS/MS scan was operated in high sensitivity mode, an accumulation time of 50 ms and a mass tolerance of 100 ppm. Former MS/MS-analyzed candidate ions were excluded for 12 sec after its first occurrence to reduce redundant peptide sequencing. The DDA data file (*.wiff) was recorded using Analyst-TF (v.1.7) software.

#### Data-independent acquisition (DIA) mode

DIA method was built using the SWATH-MS acquisition method editor. A predefined mass window width of 8 m/z with overlapping of 1 m/z for 57 transmission windows was used. A TOF MS scan was set to go through 1,715 cycles, where each cycle performs one TOF-MS scan type (250 ms accumulation time, across the 350–750 precursor mass range) acquired in every cycle for a total cycle time of ~3.15 s. MS spectra were collected from 100–1250 m/z with an accumulation time of 50 ms per SWATH window width. Nominal resolving power for MS1 and SWATH-MS2 scan are 30,000 and 15,000, respectively. The rolling collision energy was applied with the collision energy spread of 15. The DIA data file (*.wiff) was recorded by Analyst-TF (v.1.7) software.

### Spectral library generation and manual curation

A total of 16 DDA data from various urinary ELV, MV and UP fractionations were used for spectral library generation. Merge search of 16 DDA files were accomplished by Protein Pilot v.5.0, revision 4769 (Sciex) using Paragon algorithm against SwissProt *Homo Sapiens* database (v.050318, 20,328 entries with isoforms) with an automated false discovery rate and the searching parameters as followed; alkylation on cysteine by iodoacetamide, tryptic digestion, TripleTOF 5600 instrument, gel-based ID special factors, ID focus on biological modification, thorough ID search effort, and detected protein threshold [unused ProtScore (Conf)] >0.05 (10%). The standard target-decoy database searching method in the Protein Pilot software was applied to estimate false discovery rates (FDR) at the peptide spectrum match (PSM), peptides and protein levels, while the non-linear fitting method was used to determine both global and local FDR from the decoy database searching. The PSM-, peptide-, and protein-level FDR values along with the total number of expected true positives and false positives at each level were shown in Supplementary Figure S3. The search result was manually inspected for unique peptides and proteins with FDR <1% which were considered valid.

The Protein Pilot search result (*.group) was loaded onto SWATH Acquisition MicroApp v.2.0.2133 in PeakView software v.2.2 (Sciex) to generate an original spectral library. The maximum number of protein to import for spectral library generation was set as the number of proteins identified at 1% global FDR from fit. The original spectral library file (*.txt) was then exported into an Excel worksheet for manual curation.

Manual curation based on the concept of high-quality assay library^19^ was performed in Excel to remove the source of assay inconsistency and variability as followed; (i) any peptide with modification, except carboxamidomethyl (CAM) of cysteine; (ii) any peptide with missed cleavage; (iii) any peptide that not terminated by lysine or arginine (which considered as non-tryptic peptides); (iv) any peptide that identified as contamination or reversed sequences. Albumin and THP peptides which eluted at different RT covered chromatographic range were used for endogenous RT alignment across all replicates. The curated spectral library was used for SWATH data extraction.

### SWATH-MS analysis

SWATH data extraction of nine DIA files (2.5 μg/injection) was performed by SWATH Acquisition MicroApp (Sciex) using an extraction window of 5 min and the following parameters: 10 peptides/protein, 6 transitions/peptide, excluding shared peptides, peptide confidence >99%, FDR <1%, and XIC width of 0.05 Da. For SWATH data processing, the FDR calculation was performed at the peptide level by the standard target-decoy analysis that built-in the SWATH Acquisition MicroApp, and the result was demonstrated in Supplementary Table S3. Sequence information and quantitative data of 2,231 target peptides detected and quantified at FDR <1% (corresponding to 888 proteins) were shown in Supplementary Table S4. SWATH quantitative data was exported into an Excel file for further analysis.

### Data preprocessing, visualization and statistical analysis

Data was preprocessed by Total Area Sum (TAS) approach^20^ using the following equation; [TAS-normalized protein intensity = (raw SWATH intensity of a particular protein/total intensity of all measured proteins) × 10^8^]. No missing value imputation was done. Data visualization, functional annotation and statistical analysis were performed by Excel, R package MetaboAnalystR (www.metaboanalyst.ca)^21^, Heml (v.1.0.3.7)^22^, and David bioinformatics resources 6.8^23^ as appropriate, and *p*-value < 0.05 after Benjamini-Hochberg correction was considered statistically significant.

## Electronic supplementary material

Supplementary Information

Supplementary tables

## Data Availability

The mass spectrometry proteomics data, the original and curated spectral libraries and SWATH extraction data have been deposited to the ProteomeXchange Consortium (http://proteomecentral.proteomexchange.org) via the PRIDE partner repository (https://www.ebi.ac.uk/pride/archive) with the dataset identifier ProteomeXchange: PXD008891.
