# Supplementary material for: Multiplex Biomarker Screening Assay for Urinary Extracellular Vesicles Study: A Targeted Label-Free Proteomic Approach
Source: Sci Rep. 2018 Oct 9;8:15039. doi: 10.1038/s41598-018-33280-7 (PMC6177456; doi:10.1038/s41598-018-33280-7)
Supplement: Supplementary file 1 — Supplementary Information [file 41598_2018_33280_MOESM1_ESM.pdf]

Multiplex Biomarker Screening Assay for Urinary Extracellular Vesicles Study: A Targeted Label-Free Proteomic Approach

Somchai Chutipongtanate[1,2,*] and Kenneth D. Greis[1,*]

[1] UC Proteomics Laboratory, Department of Cancer Biology, University of Cincinnati College of Medicine, Cincinnati, Ohio, United States.

[2] Pediatric Translational Research Unit, Department of Pediatrics, Faculty of Medicine Ramathibodi Hospital, Mahidol University, Bangkok, Thailand.

* Shared equal contribution and co-corresponding author

Running title: SWATH-MS for urinary extracellular vesicle study

Keyword: Biomarker; Exosomes; Human urine; Microvesicles; Post-acquisition data extraction; Proteome

* Please correspondence to:

Somchai Chutipongtanate, Ph.D., M.D.
3125 Eden Avenue, Vontz Center, Room 1208, Cincinnati, Ohio 45267, USA
Tel: +1-513-5584057, Fax: +1-513-558-4454
E-mail: schuti.rama@gmail.com, chutipsi@ucmail.uc.edu

Kenneth D. Greis, Ph.D.
Professor in Cancer Biology and Director of UC Proteomics Laboratory
3125 Eden Avenue, Vontz Center, Room 2304, Cincinnati, Ohio 45267, USA.
Tel: +1-513-5587102; Fax: +1-513-558-4454
E-mail: ken.greis@uc.edu

SUPPLYMENTARY INFORMATION

Supplementary materials and methods

Nanoparticle tracking analysis (NTA)

Particle size distribution and particle concentration of ELV, MV and UP samples were evaluated using a NanoSight NS300 instrument equipped with a sample chamber and a 532 nm green laser (Malvern Panalytical Ltd., Worcestershire, UK). Each sample was diluted 500 times before injection into a flow-cell with a 1-mL sterile syringe. The scattered light of the particles was captured by a sCMOS camera and data were then analyzed by the NTA 3.1 Build 3.1.54 software (Malvern) for particle size distribution and concentration. The measurements were performed in five replicates per specimen.

Supplementary figure legends

Supplementary figure S1 online. Full-length blot results of ALIX (**a** and **d**), HSP70 (**b** and **e**) and TSG101 (**c** and **f**), corresponding to a group of cropped blot images showed in **Figure 2**.

Supplementary figure S2 online. Particle size distributions in ELVs (**a**), MVs (**b**) and UPs (**c**) and average particle concentrations of all collected fractions (**d**) were measured by nanoparticle tracking analysis. The black lines and red color-labeled bands (**a-c**) represented mean and SD in collected fractions, respectively. Note that the dominant population of particles in ELVs had a diameter of 103 nm, whereas heterogeneity in the particle size (that varied within a range of 42 – 715 nm) was observed in MVs. All experiments were performed in 5 replicates.

Supplementary figure S3 online. A summary of the PSM-, peptide-, and protein-level FDR values along with the total number of expected true positives and false positives at each level obtained from the merge search of 16 DDA files using Protein Pilot software against the UniProt *Homo sapiens* database (v.050318; 20,328 entries with isoforms).

Supplementary table legends

Supplementary table S1 online. David functional annotation of top 10 matches in Gene Ontology (GO) term, Genetic Association Database for Disease (GAD-Disease), and KEGG pathway based on the list of 1,145 proteins in the curated spectral library.

Supplementary table S2 online. SWATH extraction data of 888 proteins detected and quantified in ELVs, MVs and UP samples.

Supplementary table S3 online. The peptide-level FDR values generated by SWATH data processing of ELVs, MVs and UP samples.

Supplementary table S4 online. Sequence information and quantitative data of 2,231 target peptides, corresponding to 888 proteins detected and quantitated by SWATH analysis.

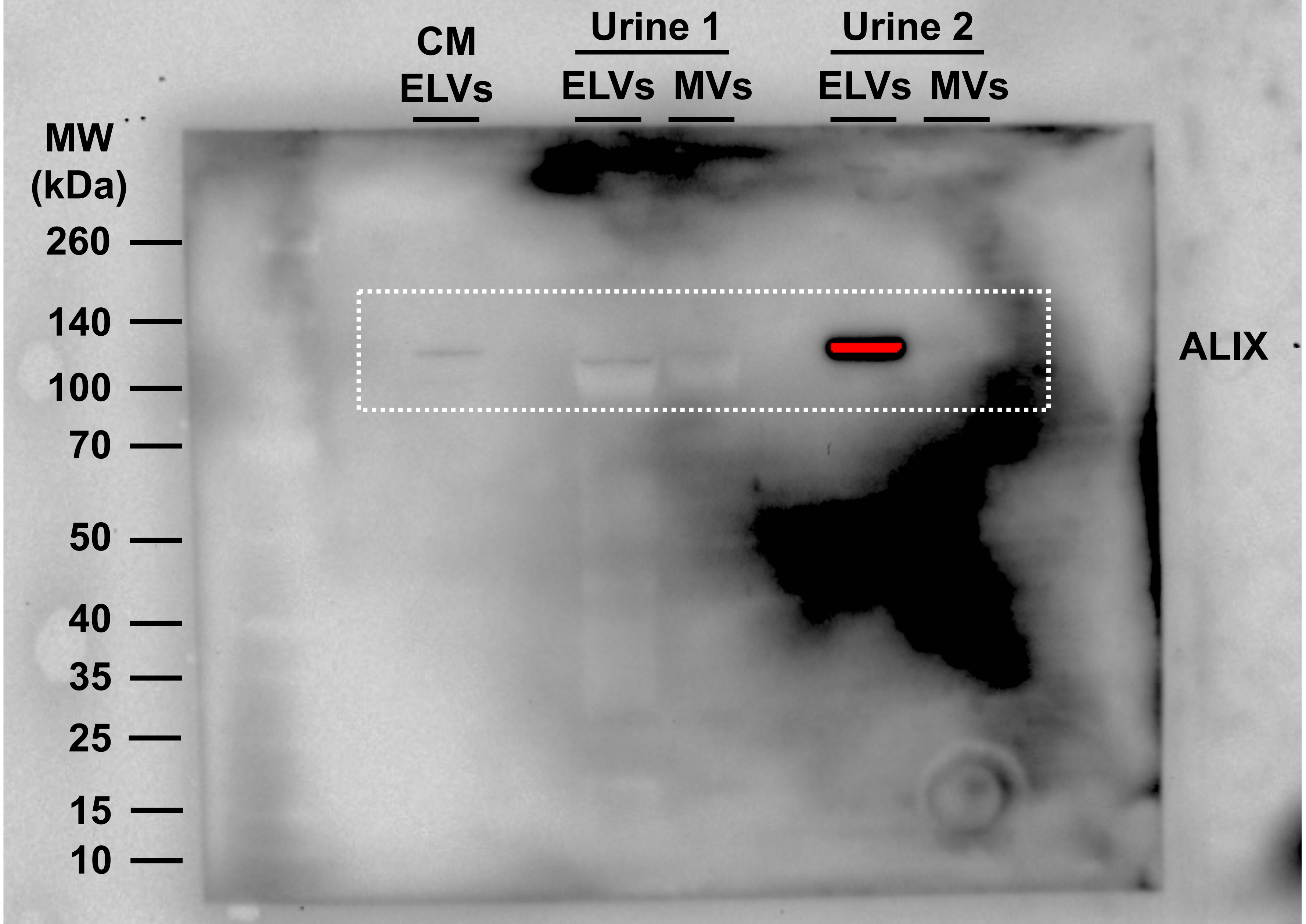

Supplementary figure S1a online

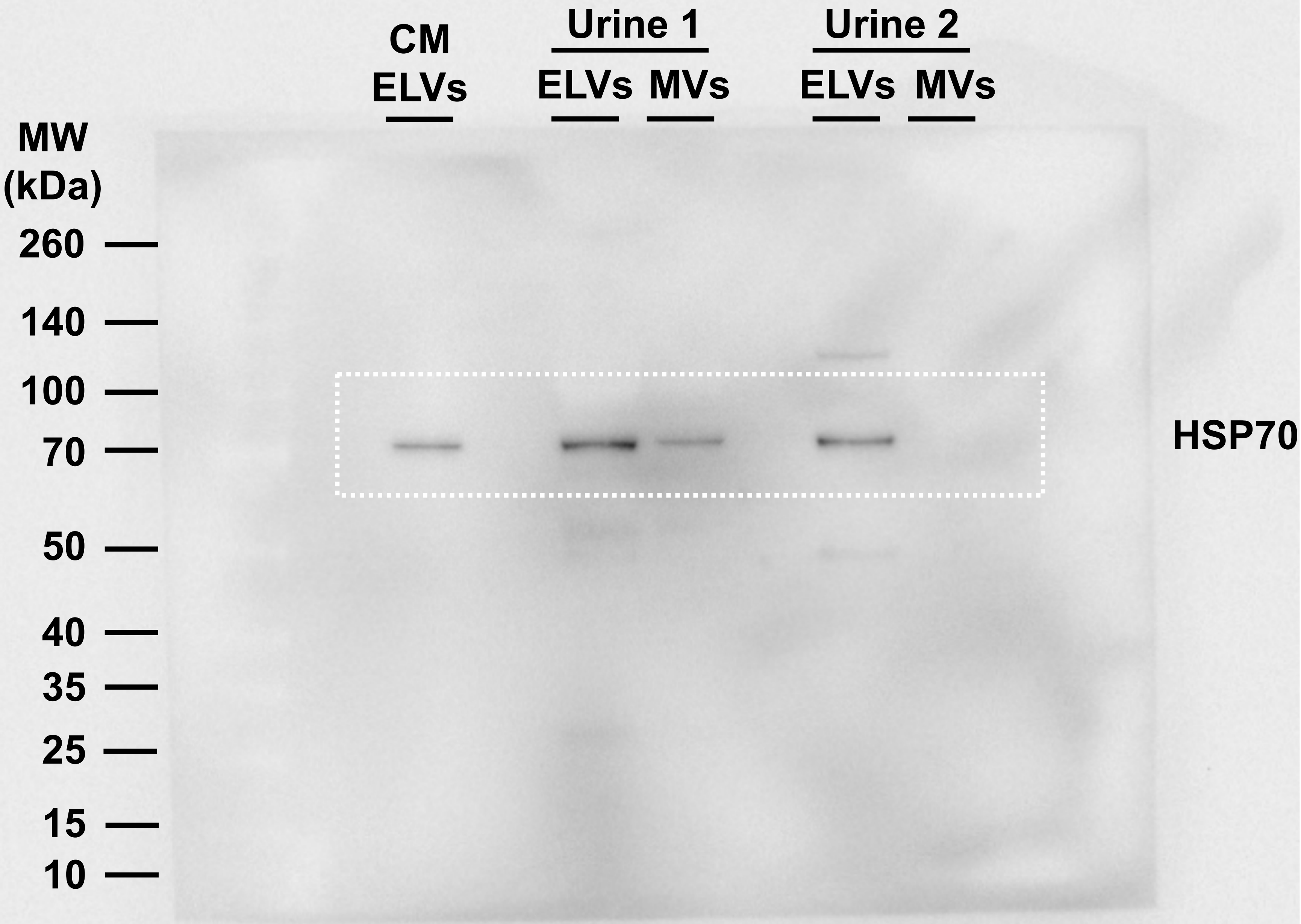

Supplementary figure S1b online

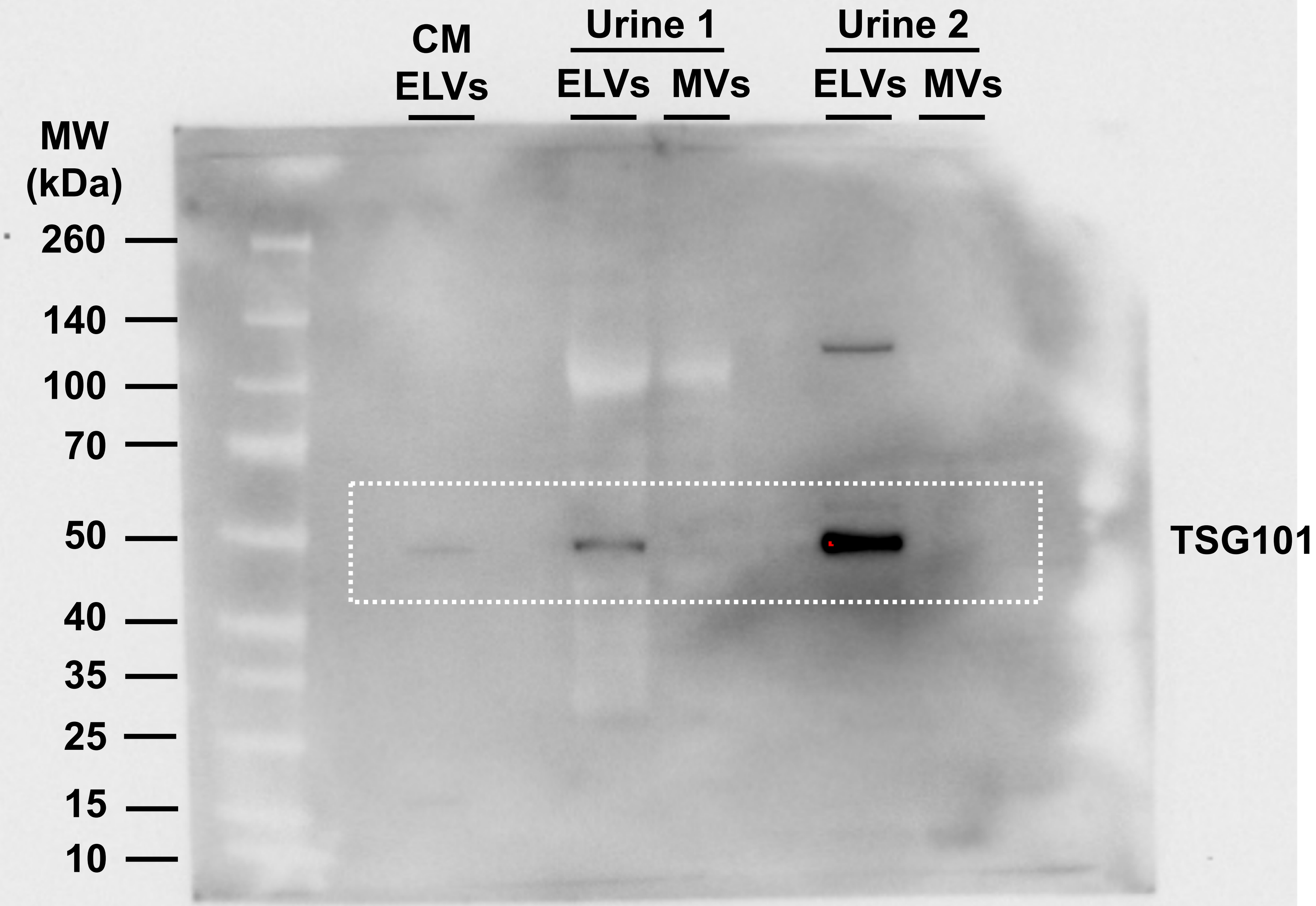

Supplementary figure S1c online

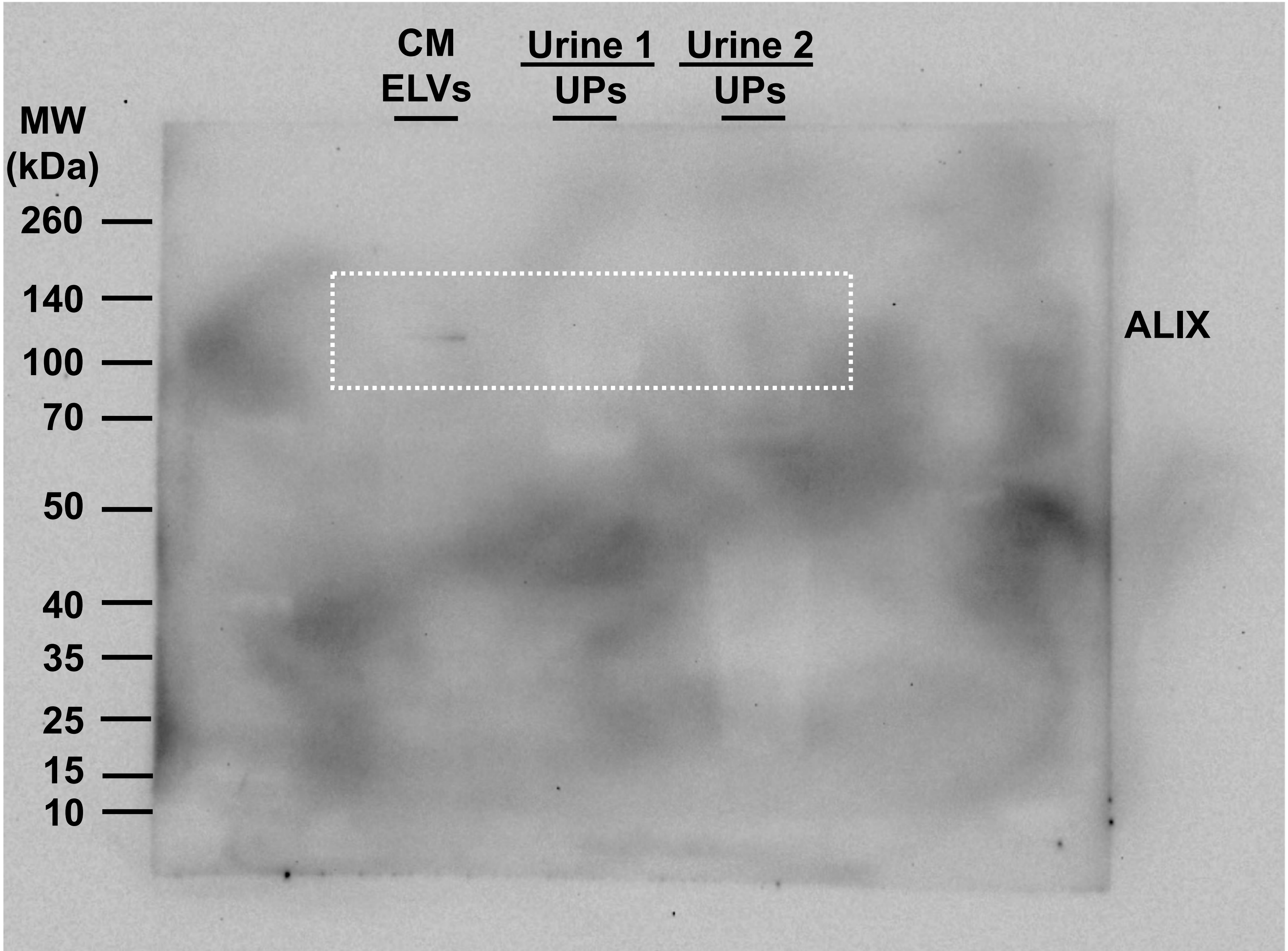

Supplementary figure S1d online

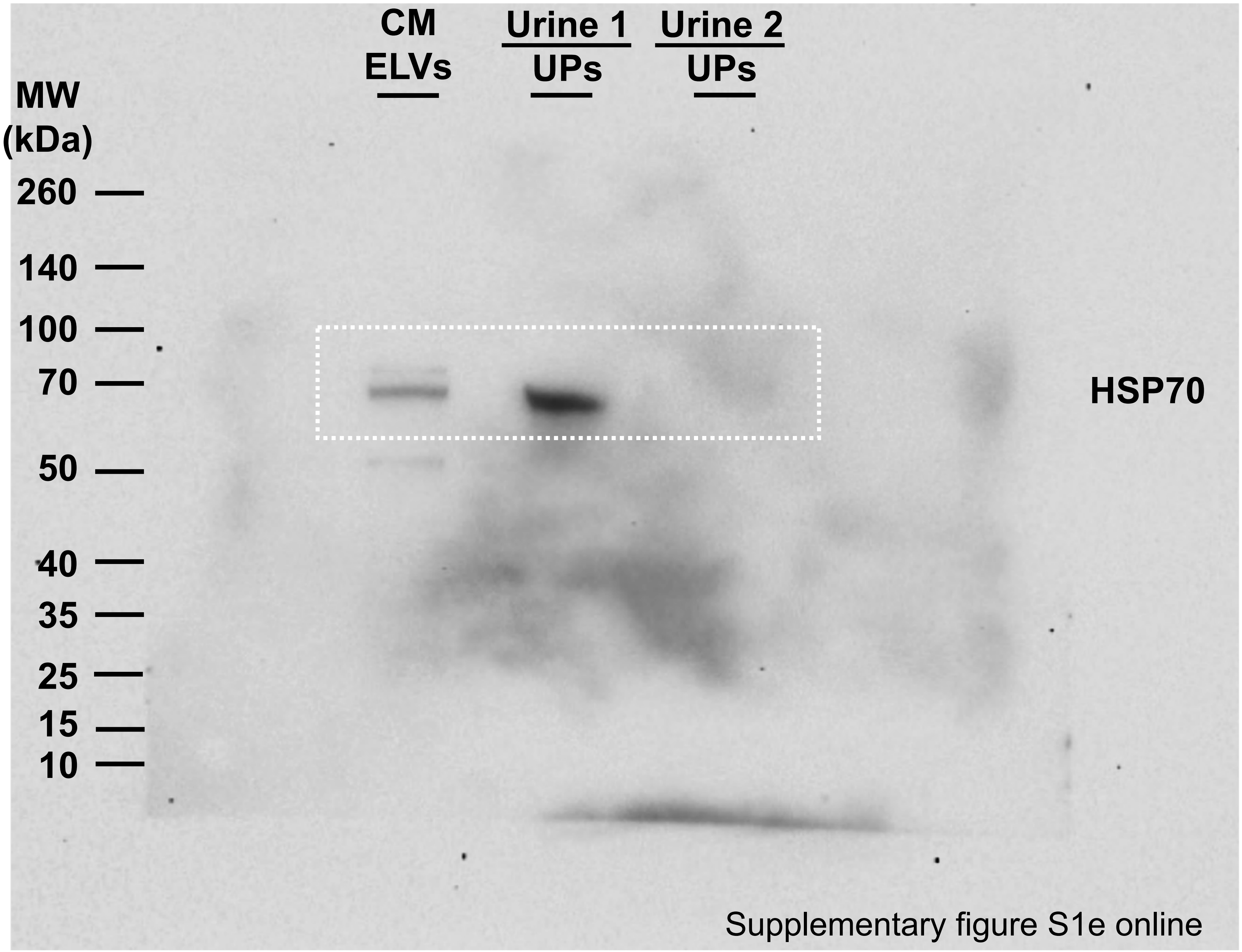

Supplementary figure S1e online

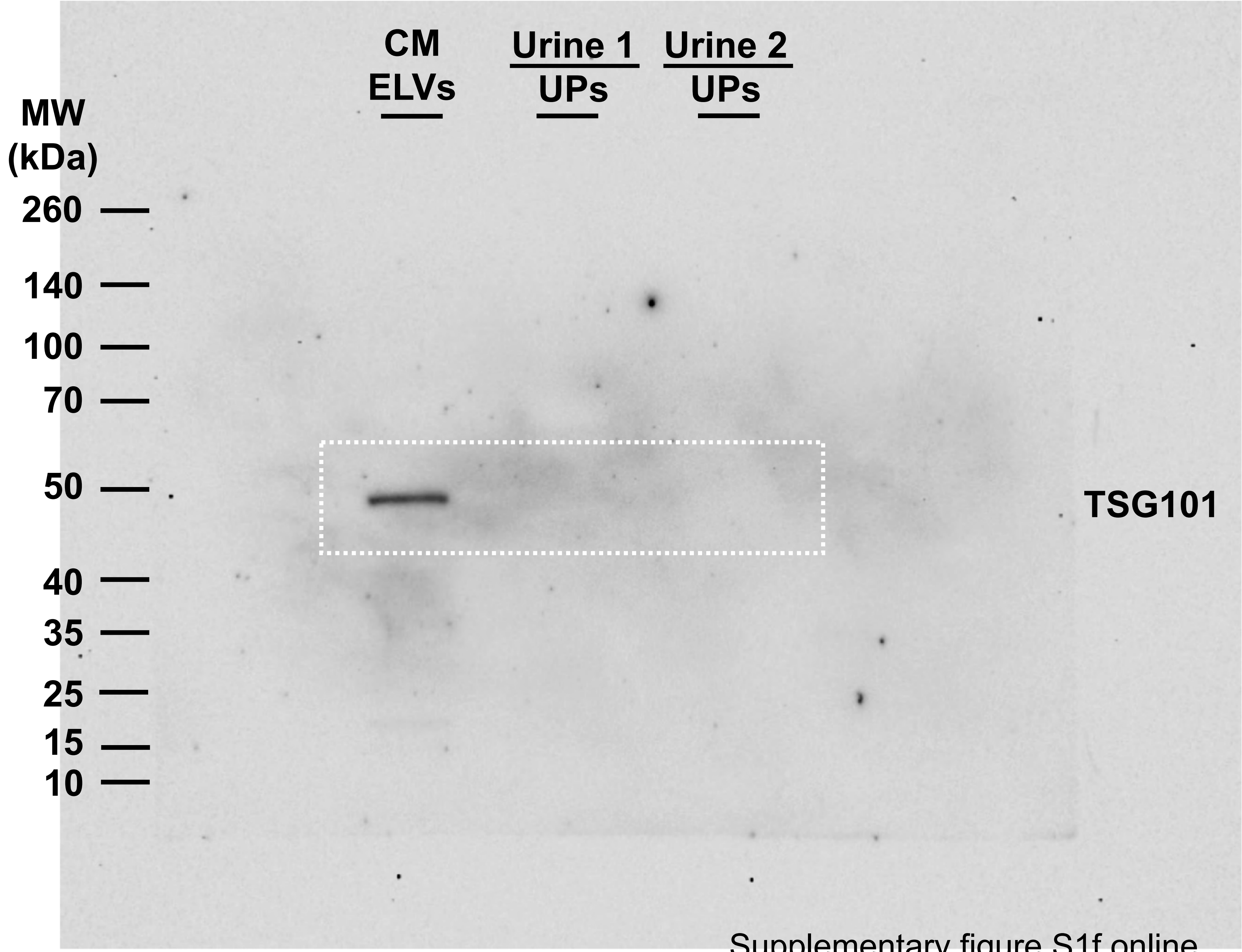

Supplementary figure S1f online

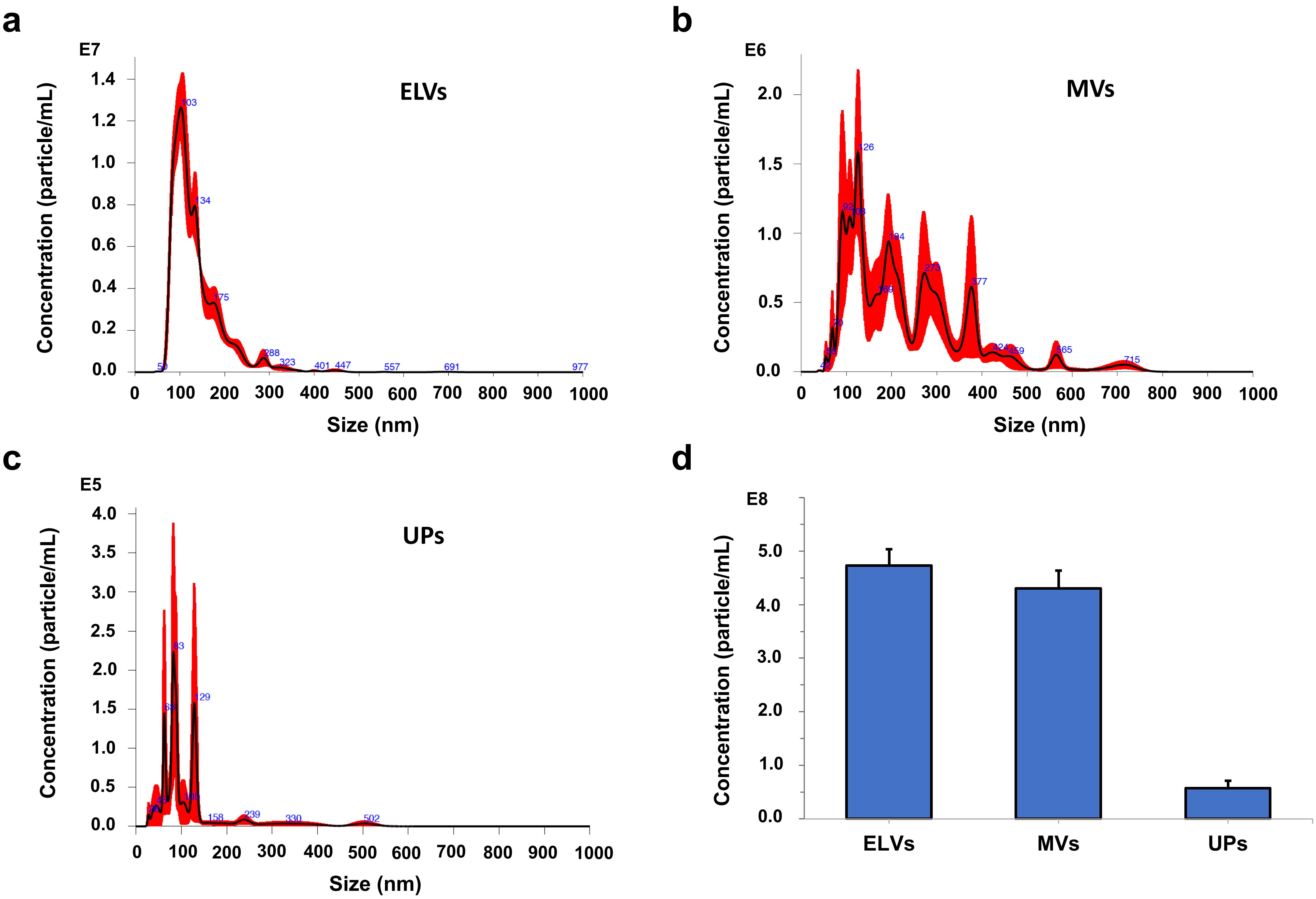

Supplementary figure S2 online

Summary of Identification Yields

Identification Yield at FDR Threshold

Data Level	FDR Type	FDR	ID Yield
Protein	Local	1%	845
		5%	1117
		10%	1195
	Global	1%	1151
		5%	1445
		10%	1588
Distinct peptide	Local	1%	11155
		5%	13684
		10%	14821
	Global	1%	14614
		5%	17902
		10%	20014
Spectral	Local	1%	105281
		5%	121521
		10%	129009
	Global	1%	131329
		5%	154593
		10%	168529

Corresponding Confidence in ProteinPilot™ Software

Data Level	FDR Type	FDR	ID Yield
Protein	Local	1%	99.9%
		5%	99.0%
		10%	95.8%
	Global	1%	98.2%
		5%	33.9%
		10%	12.9%
Distinct peptide	Local	1%	99.3%
		5%	95.4%
		10%	88.5%
	Global	1%	90.4%
		5%	42.7%
		10%	24.4%
Spectral	Local	1%	96.7%
		5%	81.3%
		10%	62.0%
	Global	1%	55.5%
		5%	13.8%
		10%	0.1%

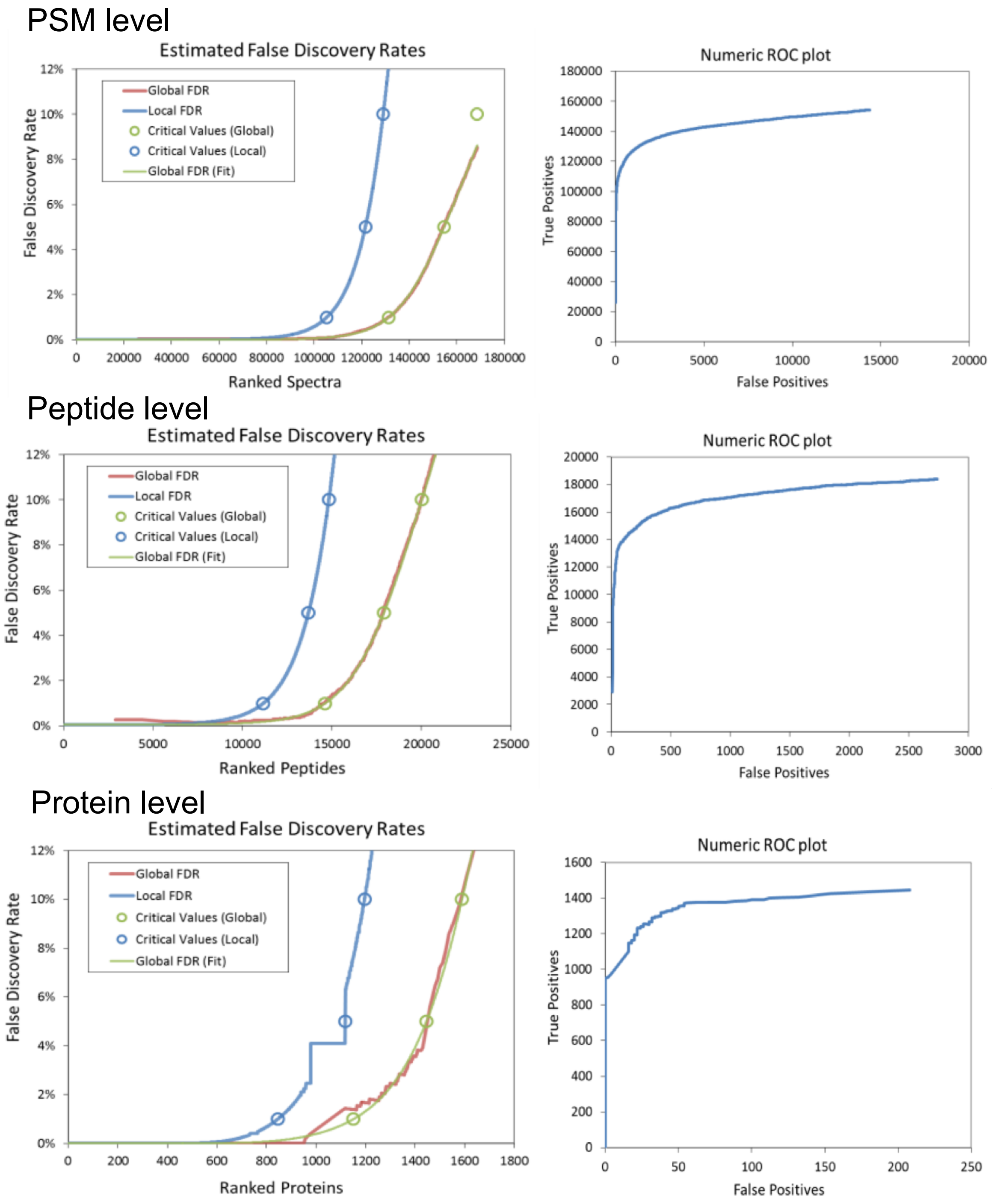

Supplementary figure S3 online
